# Re: Pre-operative COVID-19 screening for urologic patients

**DOI:** 10.1590/S1677-5538.IBJU.2021.0245

**Published:** 2021-09-15

**Authors:** Pathum Sookaromdee, Viroj Wiwanitkit

**Affiliations:** 1 Private Academic Consultant Bangkok Thailand Private Academic Consultant, Bangkok Thailand; 2 Dr. DY Patil University Pune India Dr. DY Patil University, Pune, India

To *the editor,*

We would like to share ideas on the publication “Pre-operative COVID-19

screening: a model to provide non-discretionary care for urologic patients” Ferenczi et al. (1). concluded that “Universal COVID-19screening of pre-operative patients represents aviable means to meet the needs of our patients” (1). Since COVID-19 might be asymptomatic, a COVID-19 screening is a useful preventive measure. Nevertheless, there are some important considerations. First, the cost of screening should be considered. A cost effective screening tool should be selected. Second, any test can provide a false result and limitation of sensitivity might be problematic (2). Universal preventive guidelines are still important. The practitioner has to strict follow the standard universal prevention protocols. Finally, it is necessary to set a good method for management of a positive case. If a positive result is the cause of under standardized clinical management, it is considered unethical. Since there is no present recommendation for specific therapy for COVID-19, further researches are necessary (3).

The Authors
